# Evidence of cerebellar TDP-43 loss of function in FTLD-TDP

**DOI:** 10.1186/s40478-022-01408-6

**Published:** 2022-07-25

**Authors:** Sarah Pickles, Tania F. Gendron, Yuka Koike, Mei Yue, Yuping Song, Jennifer M. Kachergus, J. Shi, Michael DeTure, E. Aubrey Thompson, Björn Oskarsson, Neill R. Graff-Radford, Bradley F. Boeve, Ronald C. Petersen, Zbigniew K. Wszolek, Keith A. Josephs, Dennis W. Dickson, Leonard Petrucelli, Casey N. Cook, Mercedes Prudencio

**Affiliations:** 1 Department of Neuroscience, Mayo Clinic, Mangurian Research Building, 4500 San Pablo Road, Jacksonville, FL 32224 USA; 2 Mayo Clinic Graduate School of Biomedical Sciences, Jacksonville, FL USA; 3 Department of Cancer Biology, Mayo Clinic, Jacksonville, FL USA; 4 Department of Neurology, Mayo Clinic, Jacksonville, FL USA; 5 Department of Neurology, Mayo Clinic, Rochester, MN USA

**Keywords:** Cerebellum, Frontotemporal lobar degeneration, Stathmin-2, TDP-43

## Abstract

Frontotemporal lobar degeneration with TDP-43 pathology (FTLD-TDP) is a neurodegenerative disease primarily affecting the frontal and/or temporal cortices. However, a growing body of evidence suggests that the cerebellum contributes to biochemical, cognitive, and behavioral changes in FTLD-TDP. To evaluate cerebellar TDP-43 expression and function in FTLD-TDP, we analyzed TDP-43 protein levels and the splicing of a TDP-43 target, *STMN2*, in the cerebellum of 95 FTLD-TDP cases and 25 non-neurological disease controls. Soluble TDP-43 was decreased in the cerebellum of FTLD-TDP cases but a concomitant increase in insoluble TDP-43 was not seen. Truncated *STMN2* transcripts, an indicator of TDP-43 dysfunction, were elevated in the cerebellum of FTLD-TDP cases and inversely associated with TDP-43 levels. Additionally, lower cerebellar TDP-43 associated with a younger age at disease onset. We provide evidence of TDP-43 loss of function in the cerebellum in FTLD-TDP, supporting further investigation into this understudied brain region.

**Supplementary Information:**

The online version contains supplementary material available at 10.1186/s40478-022-01408-6.

## Introduction

Frontotemporal dementia (FTD) is an umbrella term for syndromes presenting with deficits in behavior, executive function, and language [1, 2]. Approximately 50% of cases with frontotemporal lobar degeneration (FTLD), the neuropathological diagnosis of FTD, are characterized by cytoplasmic inclusions containing TAR DNA binding protein 43 (TDP-43) in neurons and glia, with a concomitant loss of nuclear TDP-43 [3, 4]. Consequently, TDP-43 loss of function (due to its nuclear depletion) and/or toxic gains of function caused by aggregated cytoplasmic TDP-43 are believed to underlie the neuronal susceptibility and degeneration observed in the frontal and temporal cortices in FTLD with TDP-43 pathology (FTLD-TDP) [5].

The cerebellum has historically been underappreciated in FTLD-TDP given the absence of TDP-43 inclusions and significant neurodegeneration in this neuroanatomical region. However, functional imaging studies show cerebellar involvement in cognitive abilities such as working memory, emotion, language, and attention processing [6, 7]. In addition, extensive connections exist between the cerebellum and cerebrum, including the frontal and temporal lobes [8–10]. The discovery of a hexanucleotide repeat expansion in the chromosome 9 open reading frame 72 (*C9orf72*) gene as the most common genetic cause of FTD and amyotrophic lateral sclerosis (ALS)—often referred to as c9FTD/ALS—stoked interest in the cerebellum. The cerebellum of c9FTD/ALS cases is marked by robust dipeptide-repeat protein pathology and foci of repeat RNA transcripts [11–14]. Reports that a smaller repeat expansion size in the cerebellum offers a survival advantage [15, 16], and that cerebellar poly(GP) dipeptide repeat protein levels associate with cognitive impairment [14] further support cerebellar involvement in c9FTD/ALS. Moreover, studies uncovered cerebellar transcriptome alterations [17, 18], and progressive atrophy in cerebellar subregions associated with cognitive and motor symptoms in FTLD-TDP cases with or without a *C9orf72* repeat expansion [19–24]. In aggregate, these data implicate cerebellar anomalies in FTLD-TDP, beyond direct *C9orf72*-associated pathology, leaving the causes of cerebellar dysfunction in FTLD-TDP unanswered.

Previous studies discovered that TDP-43 suppresses the inclusion of cryptic exons in numerous transcripts [25], including *STMN2* [26, 27]. We recently demonstrated that a truncated variant of *STMN2* (*tSTMN2*)*,* generated by the aberrant inclusion of a stop-codon containing cryptic exon, is detected in the central nervous system of ALS and FTLD cases with TDP-43 proteinopathy [28]. Intriguingly, in analyses of RNA sequencing datasets, *tSTMN2* was also detected in the cerebellum of some ALS and ALS/FTLD cases [28], suggesting a loss of TDP-43 splicing activity in this region.

Given the mounting evidence implicating cerebellar anomalies in FTLD-TDP, we evaluated TDP-43 splicing function and expression in the cerebellum of a cohort of well-characterized FTLD-TDP cases.

## Materials and methods

### Study design

Post-mortem brain tissue from individuals with neuropathologically confirmed FTLD-TDP and those without neuropathological features were provided by the Mayo Clinic Florida Brain Bank. All participants or their family members gave written informed consent, and all protocols were approved by the Mayo Clinic Institution Review Board and Ethics Committee. Sample size was determined based on the availability of tissue in our brain bank. A description of patient characteristics is provided Table 1.

**Table 1:** Characteristics of controls and FTLD-TDP cases

Variable	Controls N = 25	FTLD-TDP N = 95	P value
Sex			0.6644^a^
Male	13 (52.0%)	54 (56.8%)	
Female	12 (48.0%)	41 (43.2%)	
Age at disease onset (years)	NA	63.6 (44.0, 83.0)	
Disease duration (years)	NA	8.0 (2.0, 25)	
Age at death (years)	81.9 (56.6, 99.0)	73.1 (52.4, 90.4)	**0.0058**^b^
Genotype			
No mutation	NA	33 (34.7%)	
*C9orf72* repeat expansion	NA	29 (30.6%)	
*GRN* mutation	NA	33 (34.7%)	
TDP-43 subtype			
A	NA	68 (71.6%)	
B	NA	8 (8.4%)	
C	NA	15 (15.7%)	
RIN	9.7 (7.1, 10)	9.4 (7.2, 10.0)	**0.0375**^b^

The sample median (minimum, maximum) is given for continuous variables. Information was unavailable regarding age at disease onset and disease duration for 10 FTLD-TDP cases; age at death for 1 FTLD-TDP case; TDP-43 subtype for 4 FTLD-TDP cases. A Chi-squared test was used to test for differences in sex^a^, and a Mann–Whitney test was used to test for differences in age at death and RIN^b^ between control and FTLD-TDP groups. P-values < 0.05 are considered statistically significant and are marked in bold

### RNA extraction and NanoString analysis in post-mortem brain tissue

RNA was extracted from postmortem frozen cerebellum tissue using the RNAeasy Plus Mini Kit (Qiagen) per the manufacturer’s instructions. RIN was assessed using an Agilent 2100 bioanalyzer (Agilent Technologies), and only samples with an RNA integrity number (RIN, ≥ 7.0) were used. Levels of the *tSTMN2* transcript were determined using 250 ng of RNA using the NanoString PlexSet platform. Data was analyzed using transcript nSolver4.0 software (NanoString Technologies) and normalized to *hypoxanthine phosphoribosyltransferase (HPRT1)*, a housekeeping gene we have previously used to normalized transcript levels [28]. Probe sequences are as follows: *tSTMN2* 5’-AGAAGACCTTCGAGAGAAAGGTAGAAAATAAGAATTTGGCTCTCTGTGTGAGCATGTGTGCGTGTGTGCGAGAGAGAGAGACAGACAGCCTGC-3’, and *HPRT1* (NM_000194.3), 5’- CTATGACTGTAGATTTTATCAGACTGAAGAGCTATTGTAATGACCAGTCAACAGGGGACATAAAAGTAATTGGTGGAGATGATCTCTCAACTTTAACTGG-3’.

### Protein extraction and immunoblotting

Radioimmunoprecipitation assay buffer (RIPA)-soluble and urea-soluble protein fractions were obtained from postmortem cerebellar tissue, as previously described [14]. Approximately 50 mg of tissue were homogenized in cold RIPA buffer (25 mM Tris–HCl pH 7.6, 150 mM NaCl, 1% sodium deoxycholate, 1% Nonidet P-40, 0.1% sodium dodecyl sulfate, protease and phosphatase inhibitors) and then sonicated on ice. The homogenates were centrifuged at 100,000 × g for 30 min at 4 °C and the supernatant was collected. The pellets were resuspended in RIPA buffer and sonicated. The samples were then centrifuged again and extracted using 7 M urea, sonicated, and centrifuged at 100,000 × g for 30 min at room temperature. Protein concentrations of the RIPA and urea-soluble fractions were determined by Bicinchoninic acid assay (BCA) assay or Bradford assay, respectively. RIPA or urea-soluble protein was diluted in 2X Tris–glycine SDS sample buffer (Life Technologies) and reducing agent (5% beta-mercaptoethanol, Sigma Aldrich) and heat-denatured for 5 min at 95 °C. Samples were run by sodium dodecyl sulfate–polyacrylamide gel electrophoresis (SDS-PAGE) on 4–20% Tris–Glycine gels (Life Technologies) and transferred to Polyvinylidene (PVDF) membrane (Millipore). Following transfer, membranes were blocked in 5% non-fat dry milk in Tris buffered saline with Triton (TBS-T, 100 mM Tris–HCl pH 7.5, 140 mM NaCl, 0.1% Triton X-100) and incubated overnight at 4 °C with a rabbit polyclonal anti-TDP-43 antibody (1:1500, 12892-1-AP, ProteinTech) followed by a mouse monoclonal anti-glyceraldehyde-3-phosphate dehydrogenase (GAPDH) antibody (1:30,000, H86504M, Meridian Life Sciences). Membranes were then incubated with horseradish peroxidase (HRP)-conjugated secondary antibodies (1:5000; Jackson ImmunoResearch) and proteins of interest were detected by Enhanced chemiluminescence (ECL, PerkinElmer). Quantitative densitometry on soluble and insoluble protein fractions was performed using Image J and TDP-43 in RIPA-soluble protein fractions were normalized to GAPDH expression.

### Statistics

All statistical analyses were done using GraphPad Prism 9 (GraphPad Software). For each figure the type of analysis used, and the number of subjects is indicated in the figure and/or legend.

To compare *tSTMN2* RNA and TDP-43 protein in the cerebellum between controls and FTLD-TDP cases, all FTLD-TDP cases combined and the three genotypes separately, were analyzed with single-variable (unadjusted) and multivariable linear regression models (adjusted). Multivariable models were adjusted for age at death, sex and RIN for *tSTMN2* RNA, and for age at death and sex for TDP-43 protein. Both *tSTMN2* RNA and TDP-43 protein were analyzed on the base 2 logarithmic scale due to their skewed distributions. The regression coefficients (β) and 95% confidence intervals (CIs) were estimated and interpreted as the difference in the means, bases on the 2 logarithmic scale, between all FTLD-TDP cases combined or the individual genotypes and the controls (reference group). P values less than 0.0125 were considered statistically significant after adjusting for the four different statistical test that were performed for all FTLD-TDP and the separate FTLD-TDP groups (no mutation, *C9orf72* mutation carriers, *GRN* mutation carriers) vs. controls.

For associations between TDP-43 protein levels and *tSTMN2* RNA, age at disease onset, or disease duration after onset were evaluated in FTLD-TDP cases using single-variable and multivariable linear regression models. Both TDP-43 protein levels and *tSTMN2* RNA were analyzed using the base 2 logarithmic scale. The multivariable model examining TDP-43 protein levels and *tSTMN2* RNA was adjusted for age, sex and genotype. The model evaluating TDP-43 and age at disease onset was adjusted for sex and genotype, and the model assessing associations between TDP-43 protein and disease duration was adjusted for sex, age at onset and genotype. P values less than 0.0167 were considered statistically significant after adjusting for multiple comparisons.

To compare levels of soluble (RIPA-soluble) or insoluble (urea-soluble) TDP-43 protein a Mann–Whitney test was used.

## Results

### Truncated STMN2 RNA is elevated in the cerebellum of FTLD-TDP cases

**Fig. 1:**
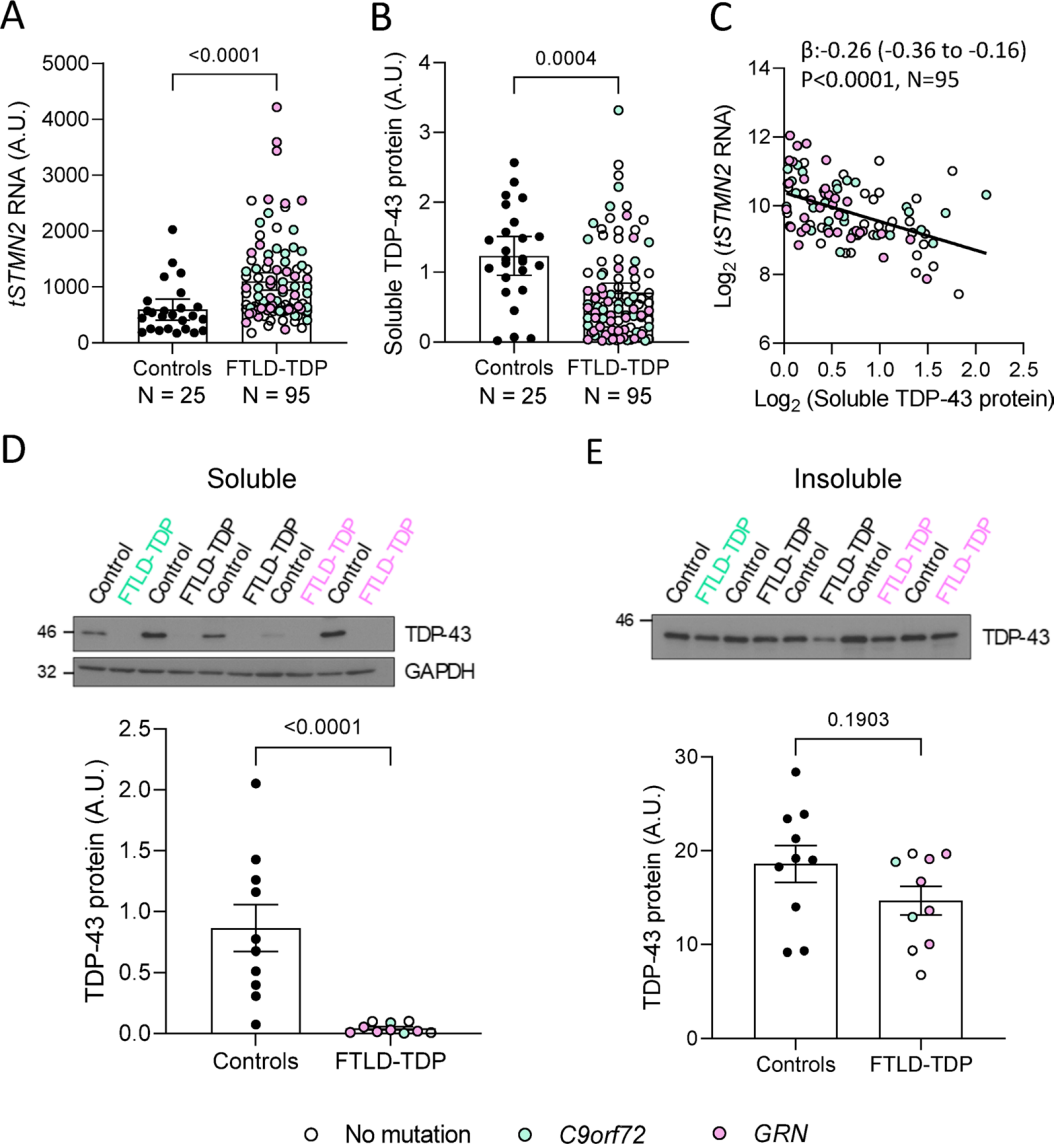
*Truncated STMN2* RNA is elevated, and soluble TDP-43 protein is decreased in the cerebellum of FTLD-TDP cases. **A**, **B** *tSTMN2* RNA and TDP-43 protein levels were measured in FTLD-TDP cases (*n* = 95) and non-neurological disease controls (*n* = 25). **A** *tSTMN2* RNA levels, as measured by NanoString PlexSet platform, are significantly elevated in FTLD-TDP cases. **B** Soluble TDP-43 protein levels, as quantified from Western blot, are significantly decreased in FTLD-TDP cases. Horizontal bars represent mean 95% confidence intervals (95% CI). P-values and 95% CI from unadjusted linear regression models are shown. Note values for linear regression models adjusted for age, sex and RIN are shown in Table 2. **C** Associations of TDP-43 protein levels with *tSTMN2* RNA levels were examined in FTLD-TDP cases. Regression coefficient (β), 95% CI and P-values from unadjusted linear regression models are shown. Values for linear regression models adjusted for age, sex and genotype are shown in Table 3. **D**, **E** TDP-43 protein levels in the cerebellum of FTLD-TDP cases (n = 8) and controls (n = 10) were measured by western blot in the RIPA-soluble (**D**) and RIPA-insoluble (**E**) fractions. A representative western-blot (*above*) and densiometric quantification (*below*) are presented. Data are presented as mean + / − SEM. P values shown resulting from Mann Whitney test

To evaluate cerebellar *STMN2* missplicing, we used the NanoString PlexSet platform to measure *tSTMN2* RNA in 95 FTLD-TDP cases ([29] with no known FTD-causing mutation, 29 with a *C9orf72* repeat expansion and 33 with a mutation in progranulin (*GRN*)) and in 25 cognitively normal controls pathologically-confirmed to have no TDP-43 pathology (see Table 1). Our control and FTLD-TDP groups were sex-matched, having similar ratios of males to females. However, individuals in our control group were significantly older and had higher RNA integrity numbers (RIN, Table 1). We used a single-variable linear regression model, referred to as the unadjusted analysis, to first determine trends in the unadjusted data and then a multivariable linear regression model, referred to as the adjusted model, to control for possible confounding factors arising from differences between control and FTLD-TDP groups. In unadjusted analysis [β:0.9352, 95% confidence interval (CI): 0.5235 to 1.347, P < 0.0001] and in analysis adjusted for age, sex and RIN (β:0.7475, 95% CI: 0.3797 to 1.115, P = 0.0001), cerebellar *tSTMN2* RNA was significantly higher in all FTLD-TDP cases combined or in each separate FTLD-TDP group when compared to controls (Fig. 1A, Table 2).

**Table 2:** Comparisons of cerebellar *tSTMN2* RNA, or soluble TDP-43 protein levels between controls and FTLD-TDP groups

	N	Median (minimum, maximum) levels	Unadjusted analysis		Adjusting for age, sex and RIN^a^	
			β (95% CI)	P-value	β (95% CI)	P-value
*tSTMN2* RNA						
Controls	25	480.6 (167.0, 2028)	0.00 (reference)	N/A	0.00 (reference)	N/A
All FTLD-TDP cases	95	825.3 (172.8, 4218)	0.9352 (0.5235 to 1.347)	** < 0.0001**	0.7475 (0.3797 to 1.115)	**0.0001**
No mutation	33	679.4 (172.8, 2542)	0.6433 (0.1641 to 1.123)	**0.0009**	0.6983 (0.2798 to 1.117)	**0.0013**
* C9orf72*	29	1000 (402.4, 2325)	1.107 (0.6132 to 1.600)	** < 0.0001**	0.8483 (0.3963 to 1.300)	**0.0003**
* GRN*	33	954.3 (233.5, 4218)	1.077 (0.5973 to 1.556)	** < 0.0001**	0.7264 (0.2618 to 1.191)	**0.0025**
TDP-43						
Controls	25	1.182 (0.01877, 2.568)	0.00 (reference)	N/A	0.00 (reference)	N/A
All FTLD-TDP cases	95	0.5004 (0.01675, 3.319)	− 0.4087 (− 0.6325 to − 0.1849)	**0.0004**	− 0.2701 (− 0.4850 to − 0.05514)	0.0142
No mutation	33	0.9068 (0.02343, 2.538)	− 0.1938 (− 0.4464 to 0.05882)	0.1314	− 0.1458 (− 0.3835 to 0.09190)	0.2268
* C9orf72*	29	0.4710 (0.01675, 3.319)	− 0.4126 (− 0.6726 to − 0.1526)	**0.0021**	− 0.2722 (− 0.5268 to 0.01782)	0.0362
* GRN*	33	0.3469 (0.01689, 1.812)	− 0.6201 (− 0.8727 to 0.3675)	** < 0.0001**	− 0.4736 (− 0.7301 to − 0.2170)	**0.0004**

β = regression coefficient; CI = confidence interval; RIN = RNA integrity number. β values, 95% CIs, and p-values result from unadjusted linear regression models or linear regression models adjusted for age, sex and, when the dependent variable was RNA, RIN^a^. β values are interpreted as the difference in the mean levels of each variable of interest between controls and the indicated groups. P-values < 0.0125 are considered statistically significant after correcting for the comparisons of *tSTMN2* RNA or soluble TDP-43 protein between controls and 4 different FTLD-TDP groups and are marked in bold

### TDP-43 protein is reduced in the cerebellum in FTLD-TDP cases and associates with elevated truncated STMN2 RNA levels

To test the hypothesis that the presence of *tSTMN2* RNA in the cerebellum was caused by suboptimal TDP-43 levels, we examined detergent-soluble TDP-43 protein levels in the cerebellum by Western blot. Compared to controls, a significant or nominally significant decrease in cerebellar TDP-43 was seen in FTLD-TDP cases in unadjusted analysis (β: − 0.4087, 95% CI: − 0.6325 to − 0.1849, P = 0.0004, Fig. 1B, Table 2) and analysis adjusted for age and sex (β: − 0.2701, 95% CI: − 0.4850 to − 0.05514, P = 0.0142, Table 2), respectively. This decrease in soluble TDP-43 appeared to be largely driven by *C9orf72* and *GRN* mutation carriers (Fig. 1B, Table 2). Further confirming the relationship between cerebellar TDP-43 protein and *tSTMN2* RNA in FTLD-TDP cases, we found that lower soluble TDP-43 significantly associated with higher *tSTMN2* in both unadjusted analysis (β: − 0.2604, 95% CI: − 0.3621 to − 0.1588, P < 0.0001, Fig. 1C, Table 3) and analysis adjusted for age, sex and RIN (β: − 0.2078, 95% CI: − 0.3136 to − 0.1021, P = 0.0002, Table 3).

**Table 3:** Associations of cerebellar TDP-43 with *tSTMN2* RNA, age at onset, and disease duration in FTLD-TDP cases

Variable	N	Unadjusted analysis		Multivariable analyses		
		β (95% CI)	P-value	β (95% CI)	P-value	Multivariable model adjustments
*tSTMN2* RNA	95	− 0.2604 (− 0.3621 to − 0.1588)	** < 0.0001**	− 0.2078 (− 0.3136 to − 0.1021)	**0.0002**	Age, sex, and genotype
Age at onset (years)	85	0.02175 (0.009906 to 0.03359)	**0.0005**	0.01624 (0.003352 to 0.02912)	**0.0142**	Sex and genotype
Disease duration (years)	85	0.02088 (− 0.003103 to 0.04487)	0.0871	0.02693 (0.004536 to 0.04932)	0.0190	Sex, age at onset and genotype

β = regression coefficient; CI = confidence interval. β values, 95% CIs and p-values are shown for associations of TDP-43 with the indicated variables from unadjusted linear regression models or linear regression models adjusted for indicated variable. P-values < 0.0167 are considered statistically significant after correcting for multiple comparisons and are marked in bold

To determine whether the decrease in soluble TDP-43 in FTLD-TDP cases was caused by its redistribution to the detergent-insoluble (RIPA-insoluble, urea-soluble) fraction, we measured cerebellar TDP-43 in soluble and insoluble fractions for a subset of FTLD-TDP cases and controls. More specifically, we selected the FTLD-TDP cases showing the most drastic depletion of soluble TDP-43 (Fig. 1D) as they would likely best reveal any appreciable shift of TDP-43 from the soluble to the insoluble fraction. As expected, based on our sample selection, soluble TDP-43 was significantly lower in FTLD-TDP cases than in controls; however, no concomitant rise in insoluble TDP-43 was observed (Fig. 1D, E). The lower TDP-43 levels suggest that overall cerebellar TDP-43 expression is decreased in FTLD-TDP.

### Lower levels of cerebellar TDP-43 associate with an earlier age at disease onset

**Fig. 2:**
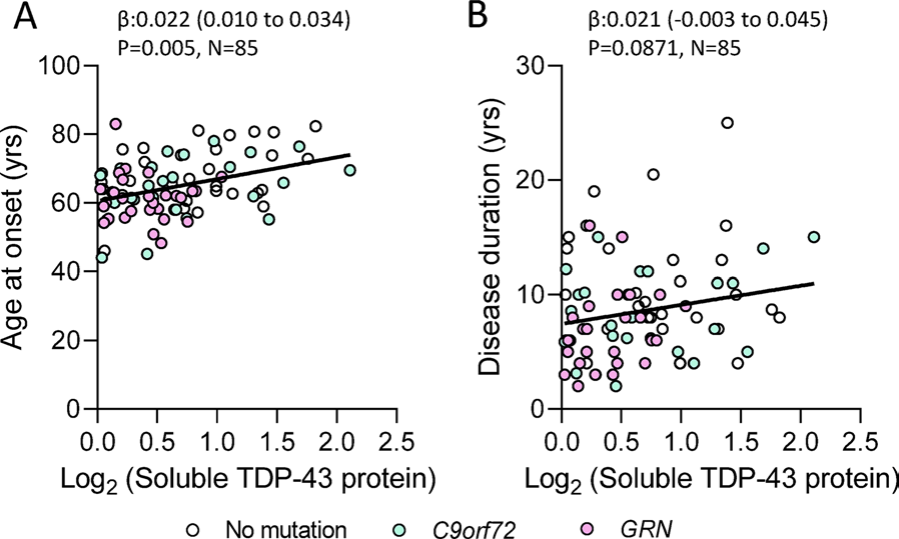
Lower levels of cerebellar TDP-43 associated with an earlier age at disease onset. Associations of TDP-43 protein levels with age at disease onset or disease duration (i.e., time from disease onset to death) were examined in FTLD-TDP cases. **A** Lower TDP-43 protein significantly associated with younger age at disease onset. **B** A non-significant trend of lower TDP-43 protein levels and shorter disease duration was observed. Regression coefficients (β), 95% confidence intervals (95% CI) and P-values from unadjusted linear regression models are shown. Values for linear regression models adjusted for age, sex and genotype are shown in Table 3

To evaluate the clinical significance of cerebellar TDP-43 depletion and elevated *tSTMN2* RNA production, we assessed whether detergent soluble cerebellar TDP-43 or *tSTMN2* RNA levels associate with age of disease onset or disease duration in FTLD-TDP cases. Lower cerebellar TDP-43 associated with a younger age at disease onset in both unadjusted analysis (β: 0.02175, 95% CI: 0.009906 to 0.03359, P = 0.0005, Fig. 2A, Table 3), and analysis adjusted for sex and genotype (β: 0.01624. 95% CI: 0.003352 to 0.02912, P = 0.0142, Table 3). Although TDP-43 protein levels did not associate with disease duration in unadjusted analysis (β: 0.02088, 95% CI: − 0.003103 to 0.04487, P = 0.0871, Fig. 2B, Table 3), a trend of lower TDP-43 and shorter disease duration was observed in analysis correcting for age, sex and genotype (β: 0.02693, 95% CI: 0.004536 to 0.04932, P = 0.0190, Table 3). Higher levels of cerebellar *tSTMN2* RNA significantly associated with an earlier age of disease in unadjusted analysis (β: − 0.02638, 95% CI: − 0.04788 to − 0.004882, P = 0.0168, Additional file 1: Table S1) and trended toward significance in analysis adjusted for RIN and sex (β: − 0.02056, 95% CI: − 0.04029 to − 0.0008248, P = 0.0414, Additional file 1: Table S1). Levels of *tSTMN2* RNA did not associate with disease duration in either unadjusted (β: − 0.01976, 95% CI: − 0.06213 to 0.02260, P = 0.3562, Additional file 1: Table S1) or analysis adjusted for age, sex, and RIN (β: − 0.01474, 95% CI: − 0.05858 to 0.02910, P = 0.5052, Additional file 1: Table S1). In aggregate, our data suggest that TDP-43-mediated dysfunction in the cerebellum contributes to FTLD-TDP pathogenesis.

## Discussion

We found decreased TDP-43 protein levels, increased *tSTMN2* RNA levels, and a significant inverse association between them in the cerebellum of FTLD-TDP cases. To our knowledge, this is the first report of TDP-43 loss of function in the cerebellum, highlighting that the consequences of a loss of TDP-43 splicing activity may be more extensive than previously thought. Several studies have found dysregulation of gene expression and splicing in the cerebellum of FTLD/ALS cases [15–18, 30, 31], with one study reporting extensive overlap between gene expression changes in the temporal and frontal cortices and the cerebellum [31]. Similarly, in Alzheimer’s disease and progressive supranuclear palsy, transcriptomic changes detected in the temporal cortex were preserved in the cerebellum [32]. Together, these studies suggest a common mechanism may account for the shared transcriptomic alterations found in distinct brain regions for a given disease. Our data suggest loss of TDP-43 protein levels, and therefore TDP-43 function, may drive these transcriptomic changes in FTLD-TDP. In support of cerebellar TDP-43 dysfunction, 70 genes with differential transcript usage, including polyadenylation, promoter usage and splicing were also identified as targets of TDP-43 [31]. Similarly, TDP-43 was recently found to repress the inclusion of a cryptic exon harbored in the *UNC13A* gene [33, 34]. The *UNC13A* transcript with cryptic exon was detected in the frontal and temporal cortices of FTLD/ALS cases with TDP-43 pathology and was also found sparingly in the cerebellum [33, 34].

That we find splicing defects in the cerebellum, a region without significant TDP-43 inclusions, suggest that TDP-43 loss of function is uncoupled from pathology. Reports documenting nuclear TDP-43 clearance in the absence of overt TDP-43 inclusions in regions with TDP-43 pathology thus bear mentioning [29, 35–38]. It is also noteworthy that TDP-43 nuclear depletion, accompanied by brain atrophy and *C9orf72*-related pathology, were observed in the temporal lobe of a *C9orf72* repeat expansion carrier following surgery for epilepsy [38]. Subsequent post-mortem analysis years later, following the onset of FTD symptoms, revealed TDP-43 inclusions in this individual, suggesting that loss of nuclear TDP-43 is an early pathological event [38]. Further, neurons depleted of TDP-43 in the absence of TDP-43 inclusions were reported to have degenerate morphologies, suggesting that TDP-43 loss of function alone can contribute to neurodegeneration [37, 38]. TDP-43-regulated cryptic exon splice variants were found to accumulate in the hippocampi of Alzheimer’s disease brains containing TDP-43-depleted neurons without TDP-43 inclusions [29]. Likewise, *tSTMN2*, the *UNC13A* variant with the cryptic exon and other alternatively spliced genes were detected in TDP-43 negative nuclei isolated from FTD/ALS cases [34, 39]. Taken together, these reports, combined with our present cerebellar data, underscore that TDP-43 nuclear clearance and loss of function can occur independently of inclusion formation and may be an early event in TDP-43 proteinopathy.

We noted that loss of soluble TDP-43 protein was more pronounced in the cerebellum of mutation carriers with the decrease in non-mutation carriers failing to reach statistical significance. However, each FTLD-TDP group (i.e., non-mutation carriers, *C9orf72* expansion carriers or *GRN* mutation carriers) showed a significant accumulation of *tSTMN2* RNA arguing that even a minor change in TDP-43 levels results in aberrant *STMN2* splicing. These data suggest that *tSTMN2* and other cryptic exon-containing transcripts normally suppressed by TDP-43 are sensitive markers of TDP-43 loss of function, thus providing a rationale to study their possible utility as biomarkers for distinguishing patients with FTD caused by TDP-43 proteinopathy vs. tauopathy—an endeavor of great importance to the FTD field.

We additionally observed that lower levels of cerebellar TDP-43 associated with an earlier age at disease onset, suggesting that maintaining cerebellar TDP-43 levels may be protective in FTLD-TDP. We also noted a trend of higher *tSTMN2* RNA associating with lower age at disease onset, in agreement with our earlier study finding a significant association between *tSTMN2* in the frontal cortex and disease onset in a larger FTLD-TDP cohort [28]. In combination with previously reported transcriptomic and splicing changes [17, 18, 31], and imaging studies demonstrating gross cerebellar atrophy [19–24], these findings strongly support a cerebellar contribution to FTLD-TDP pathogenesis.

## Conclusions

Overall, we conclude that TDP-43 loss of function may be more pervasive than initially appreciated, and that further study of the role of the cerebellum in the pathogenesis of FTLD-TDP is warranted.

## Supplementary Information

**Additional file 1: Table S1.** Associations of cerebellar tSTMN2 RNA with age at onset, and disease duration in FTLD-TDP cases.

## Data Availability

The data used in this study are available from the corresponding authors upon request.

## Abbreviations

ALS: Amyotrophic lateral sclerosis
BCA: Bicinchoninic acid assay
C9orf72: Chromosome 9 open reading frame 72
c9FTD/ALS: C9orf72-mediated FTD/ALS
CI: Confidence interval
ECL: Enhanced chemiluminescence
FTD: Frontotemporal dementia
FTLD: Frontotemporal lobar degeneration
FTLD-TDP: FTLD with TDP-43 pathology
GAPDH: Glyceraldehyde-3-phosphate dehydrogenase
GRN: Progranulin
HRP: Horseradish peroxidase
HPRT1: Hypoxanthine Phosphoribosyltransferase
PVDF: Polyvinylidene
RIN: RNA integrity number
RIPA: Radioimmunoprecipitation assay buffer
SDS-PAGE: Sodium dodecyl sulfate–polyacrylamide gel electrophoresis
STMN2: Stathmin-2
TDP-43: TAR DNA binding protein 43
TBS-T: Tris buffered saline with Triton
tSTMN2: Truncated STMN2
UNC13A: Unc-13 Homolog A
