# Supplementary material for: Evidence of cerebellar TDP-43 loss of function in FTLD-TDP
Source: Acta Neuropathol Commun. 2022 Jul 25;10:107. doi: 10.1186/s40478-022-01408-6 (PMC9310392; doi:10.1186/s40478-022-01408-6)
Supplement: Supplementary file 1 — Additional file 1: Table S1. Associations of cerebellar tSTMN2 RNA with age at onset, and disease duration in FTLD-TDP cases. [file 40478_2022_1408_MOESM1_ESM.docx]

**Table S1: Associations of cerebellar *tSTMN2* RNA with age at onset, and disease duration in FTLD-TDP cases**

|  |  | Unadjusted analysis | | Multivariable analyses | | | |
| --- | --- | --- | --- | --- | --- | --- | --- |
| Variable | N | β (95% CI) | P-value | β (95% CI) | P-value | Multivariable model adjustments |  |
| Age at onset (yrs) | 85 | -0.02638 (-0.04788 to -0.004882) | **0.0168** | -0.02056 (-0.04029 to -0.0008248) | 0.0414 | RIN and sex |  |
| Disease duration (yrs) | 85 | -0.01976 (-0.06213 to 0.02260) | 0.3562 | -0.01474 (-0.05858 to 0.02910) | 0.5052 | Sex, age, and RIN |  |
| β=regression coefficient; CI=confidence interval. β values, 95% CIs and p-values are shown for associations of *tSTMN2* with the indicated variables from unadjusted linear regression models or linear regression models adjusted for indicated variable. P-values < 0.025 are considered statistically significant after correcting for multiple comparisons. | | | | | | | |
